# Objective Assessment of Cerebellar Ataxia: A Comprehensive and Refined Approach

**DOI:** 10.1038/s41598-020-65303-7

**Published:** 2020-06-11

**Authors:** Bipasha Kashyap, Dung Phan, Pubudu N. Pathirana, Malcolm Horne, Laura Power, David Szmulewicz

**Affiliations:** 1 Networked Sensing and Control Lab, School of Engineering, Deakin University, Waurn Ponds, Victoria, Australia; 2 Florey Institute of Neuroscience and Mental Health, Parkville, Victoria, Australia; 3 Balance Disorders and Ataxia Service, Royal Victorian Eye and Ear Hospital, St Andrews Place, East Melbourne, Victoria, Australia; 4 Cerebellar Ataxia Clinic, Alfred Hospital, Prahran, Victoria, Australia

**Keywords:** Biotechnology, Computational biology and bioinformatics, Neuroscience, Health care, Medical research, Neurology, Engineering, Mathematics and computing

## Abstract

Parametric analysis of Cerebellar Ataxia (CA) could be of immense value compared to its subjective clinical assessments. This study focuses on a comprehensive scheme for objective assessment of CA through the instrumented versions of 9 commonly used neurological tests in 5 domains- speech, upper limb, lower limb, gait and balance. Twenty-three individuals diagnosed with CA to varying degrees and eleven age-matched healthy controls were recruited. Wearable inertial sensors and Kinect camera were utilised for data acquisition. Binary and multilabel discrimination power and intra-domain relationships of the features extracted from the sensor measures and the clinical scores were compared using Graph Theory, Centrality Measures, Random Forest binary and multilabel classification approaches. An optimal subset of 13 most important Principal Component (PC) features were selected for CA-control classification. This classification model resulted in an impressive performance accuracy of 97% (F1 score = 95.2%) with Holmesian dimensions distributed as 47.7% Stability, 6.3% Timing, 38.75% Accuracy and 7.24% Rhythmicity. Another optimal subset of 11 PC features demonstrated an F1 score of 84.2% in mapping the total 27 PC across 5 domains during CA multilabel discrimination. In both cases, the balance (Romberg) test contributed the most (31.1% and 42% respectively), followed by the peripheral tests whereas gait (Walking) test contributed the least. These findings paved the way for a better understanding of the feasibility of an instrumented system to assist informed clinical decision-making.

## Introduction

Cerebellar Ataxia (CA) is the term for the motor signs that result from cerebellar dysfunction. There are many causes of cerebellar dysfunction that result in CA, including various neurodegenerative conditions, lesions caused by stroke, Multiple Sclerosis (MS), tumours, trauma and toxins such as alcohol. CA can affect most aspects of movements, including balance, gait, speech, limb movements and eye movements. There is a broad relationship between the region of the body affected by ataxia and the region of the cerebellum affected by pathology: hemisphere lesions result in limb or appendicular ataxia whereas truncal and gait ataxia predominate with midline lesions. Various tests were developed to identify CA in the relevant body part: these were described over 100 years ago and, in the manner of the time, Greek or Latin terms such as dysdiadochokinesia, dysmetria and dysynergia^1^ were used to describe the disordered movements. The same tests are used today and require an experienced clinician to identify and quantify the severity of the abnormality. Consequently, the tests were formalized in scales such as the Scale of the Assessment and Rating of Ataxia (SARA) to improve consistency. The tests themselves were originally developed by Holmes and others^2,3^ to emphasise the motor dysfunction produced by CA in various body parts, but as early as the 1920’s, Holmes recognised that underlying these tests have some fundamental irregularities in speed/timing, accuracy, rhythmicity and stability.

Our overall aim is to construct superior tests for cerebellar disease for use by non-subspecialist clinicians; for deep phenotyping and identifying of biomarkers for possible CA treatment trials. In earlier studies^4–11^, we demonstrated that our instrumented devices were able to accurately emulate each of the individual bedside tests of cerebellar function and to go beyond this by grading impairment severity. The research outlined in this paper aimed to produce a composite score of CA (based on a reduced number of our instrumented tests). This composite score may prove useful in differentiating one genotype from another. By way of analogy, this process is akin to the role of formal vestibular function testing in its ability to identify both peripheral and central vestibular impairment (albeit in a much simpler dichotomy than the aim of our work in CA deep phenotyping).

While wearable sensors are increasingly being explored as measurement tools for disability in neurological diseases like Parkinson’s disease^12,13^ and MS^14^, studies are still lacking in CA^15,16^ with the potential of delivering a comprehensive, quantifiable, objective and meaningful measure of neurological function covering the 5 domains- speech, upper limb, lower limb, balance and gait. Our earlier studies^4–11^ examined data from wearable sensors while subjects with CA and normal subjects (controls) performed one or more of the standard bedside tests for CA, pertaining to any one of the 5 domains. Our aim was to use the features extracted from this data to build statistical models that could accurately predict the presence and severity of CA as measured by the SARA and other similar scales. These studies reviewed as a whole indicated that (i) each bedside test can be accurately modelled; (ii) the dominant features driving each model relate to one or more of these fundamental “Holmesian” irregularities; (iii) in case of more than one bed side test (*e.g*. for appendicular CA) there was overlapping and probably redundant information; (iv) features contributing significantly to correlate with clinical assessment were not always recognised as clinically important (*e.g*. stability was a prominent feature in the data set required to model dysdiadochokinesia although it is no more recognised as important by clinicians). The key conclusions are however, that each of the clinical tests demonstrate, to a greater or lesser degree, irregularities in the rate, rhythm, amplitude and force of movements, especially at initiation and termination of motion.

In the present study we used the results obtained in the previous CA assessments in speech^4,6^, upper limb^7–9^, lower limb^7,8^, balance^10^ and gait^11^ domains. The aim was to investigate differences between controls and CA subjects. The participants were made to wear inertial sensors simultaneously while they were being assessed for their SARA scores by the clinicians. The objectives of this study were to:

1. Assess CA in the 5 domains using nine instrumented tests based on SARA.
2. Classify the motor dysfunction of CA as measured by instrumentation into four dimensions *viz*., accuracy, timing, rhythmicity and stability based on Holmes’ definitions.
3. Investigate the intra-domain relationships and the relative importance of tests and features.
4. Based on the feature importance, identify the optimal subset of tests that contribute most to the performance accuracy in distinguishing between controls and CA subjects. In addition, investigate domain wise, test wise and feature wise contribution to the four CA dimensions.
5. Perform multilabel classification to identify CA manifestations in one or multiple domains.

Both for clinical trials and when disease modifying therapies are available, detecting the signs of emergent CA as early as possibly is important. Having developed these measurement systems, one of the next steps is to address this very question. The process of reaching this point however first required developing algorithms which could distinguish between normal and abnormal. Implicit in this approach is the need to understand the “normal” range and not assume that all controls are identical. This is essential in detecting emergent ataxia.

## Methods

### Comprehensive objective assessment (COA) system

**Figure 1:**
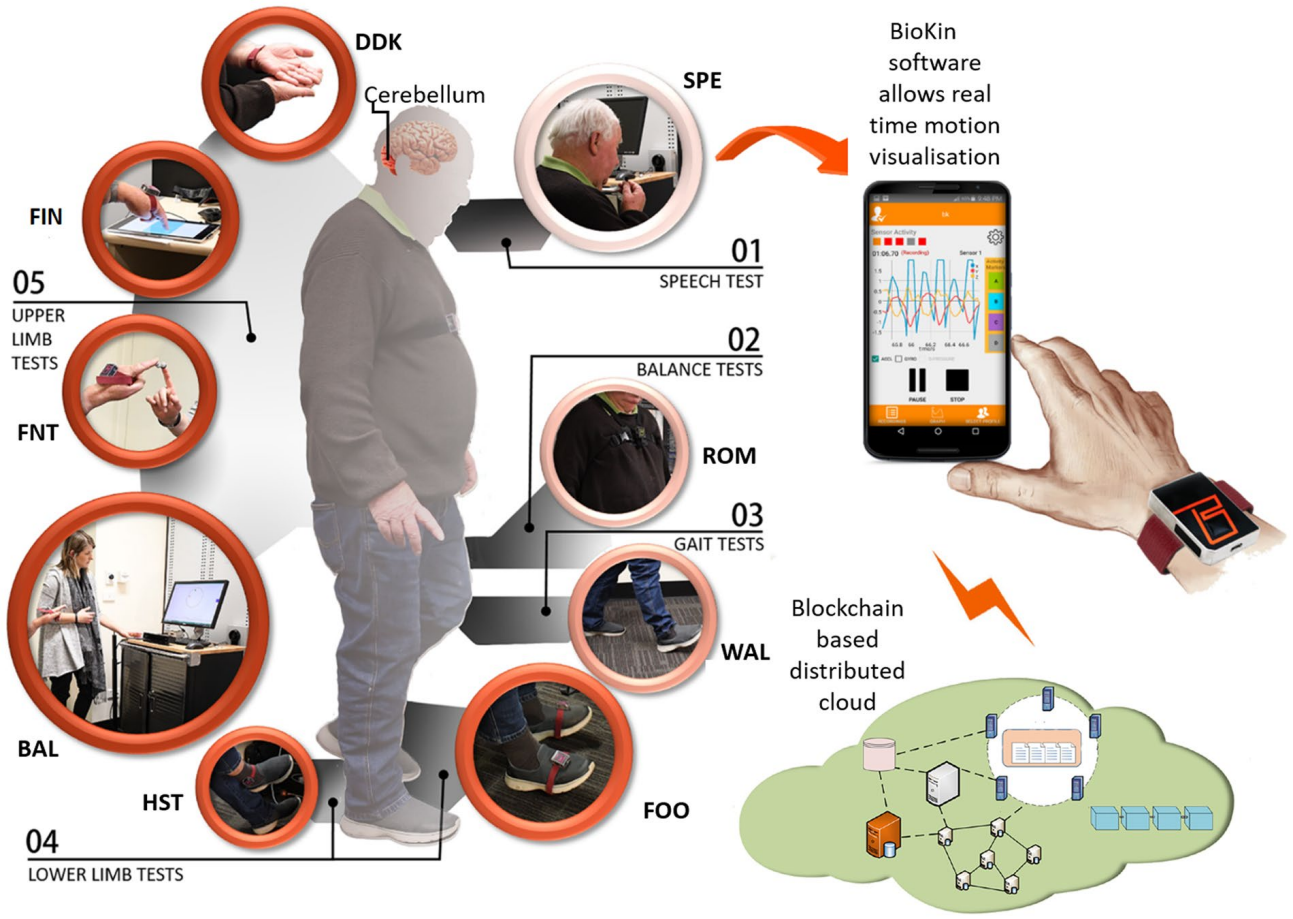
*BioKin* cloud based real time motion capture sensor platform designed for objective assessment of ataxic movements ranging from speech to lower body kinematics. Repeated syllable utterance (SPE), stance/romberg (ROM), gait (WAL), foot tapping (FOO), heel-shin (HST), ballistic tracking/finger-chase (BAL), finger-nose/nose-finger (FNT), rhythmic finger tapping (FIN), dysdiadochokinesia (DDK) are the instrumented tests.

In this study, we used *BioKin*^17^, a cloud based real time motion capture sensor platform to perform a comprehensive objective assessment of CA. *BioKin*^17^ is a wireless wearable device with an embedded tri-axial accelerometer (Model chipset “MPU-9150” from InvenSense, Inc., San Jose, CA, USA) and an IEEE802.11b/g/n/wireless communication interface running on a 32-bit ARM processor. The *BioKin* sensor system developed by Networked Sensing and Control Lab, Deakin University, can interact with an Android mobile application to capture complex movements of a human body in real time, as illustrated in Fig. 1. It is optimised to reduce settling effects and sensor drift problems by eliminating board-level cross-axis alignment errors between each inertial sensor^17^. This sensor was bench marked against a conventional multiple camera based optical motion tracking system (Vicon system, T40S, Oxford, UK), a high precision bench marking system^18^. *BioKin* captured the gyroscope and acceleration data in the three-dimensional (3D) Cartesian coordinates at a sampling rate of 50 Hz. The CA assessment was performed through the following steps:

1. *Motion Inputs* generated by nine instrumented tests that mimic the nine standard bedside clinical tests of CA covering the 5 domains.
2. These are *captured* by sensors and visualised with a supporting application in a smartphone.
3. Wireless transmission to a blockchain based distributed *cloud* network^19^ where diagnostic and assessment *algorithms* are applied.
4. Data analysis results are transformed into a *clinically relevant format*.

A pictorial representation of the sensor platform is illustrated in Fig. 1.

#### Motion input

Subjects were made to perform nine standard clinical tests; repeated syllable utterance (SPE), rhythmic finger tapping (FIN), finger-nose/nose-finger (FNT), dysdiadochokinesia (DDK), ballistic tracking/finger-chase (BAL), heel-shin (HST), foot tapping (FOO), stance/romberg (ROM) and gait (WAL). The tasks are performed inline with the instructions specified in SARA that cover all the 5 domains. To avoid any confounding factors due to sway while performing the tests in the sitting position, the participants were provided axial support *i.e*. seated comfortably in an upright sitting position. The backrest support of the chair was angled at 90–100 degrees with adequate lumber support in line with lower back. Both the right and left limbs were assessed.

#### Data acquisition

The COA System utilized Inertial Measurement Units (IMUs) equipped in the *BioKin* system in seven of the tests to capture translational and rotational kinematics in orthogonal axes via accelerometers and gyroscopes. A Microsoft Kinect V2 camera equipped with a 23 inch monitor, and mini PC with an Intel core i5 processor was also used in one of the tests for the marker based motion capture and to obtain the absolute position information.

#### Cloud based algorithms

The recorded data are then transferred to the blockchain based distributed cloud network^19^ via the wireless connection for subsequent data processing enabling physicians to acquire severity scores. MATLAB (R2019a, MathWorks) and Python environments processed the data transmitted through wireless means.

#### Clinical output

The captured data is visualised through the android based smart phone application, *BioKin* that generates evaluation reports and severity scores as a result of the cloud based data processing.

In our COA System, the average temporal expenditure in acquiring the data via motion capture from each of the 6 peripheral tests (DDK, FNT, FIN, BAL, FOO, HST) is less than 15 seconds. The balance test (ROM) takes less than 30 seconds; the gait test(WAL) takes less than 90 seconds while the speech test (SPE) takes less than 5 seconds to acquire data. Hence, the average temporal expenditure to acquire data and generate a cumulative test result (using cloud based algorithms) for a single patient is approximately under 215 seconds and 5 seconds respectively. The test set typically takes up to 30 seconds.

### Experimental design

#### Participants

For an in-depth analysis of abnormalities attributed to timing, stability, accuracy and rhythmicity in motor movements, speech and kinematic data was recorded from 34 subjects whose native language was English. Twenty-three were previously diagnosed with a Cerebellar Ataxia (CA) due to a neurodegenerative disorder and attended the Neurology clinic at the Royal Victorian Eye and Ear Hospital (RVEEH) or Alfred Hospital in Melbourne. Eleven normal subjects (controls) were volunteers without any history of neurological conditions or other speech disorders. Summary of the cohort statistics are given in Table 1. None of the participants (controls and CA) had undergone any rehabilitation program prior to this clinical investigation. None of the participants (controls and CA) had undergone any rehabilitation program prior to this clinical investigation. A review of the literature^20–22^ revealed that age of onset, age of diagnosis and other demographic factors do not influence age and gmoender-related to ataxia. As our study was confined to an adult-onset ataxia cohort, strict adherence to the age and gender matched criteria was not feasible.

**Table 1:** Clinical Characterisation of the enrolled participants.

Characteristics		Patients (n = 23)	Controls (n = 11)
Age (years)		65 ± 11(41–80)	58 ± 12(54–71)
Age of Diagnosis (years)		59 ± 9.7(40–69)	—
Length of Diagnosis (years)		11.7 ± 6.3(40–69)	—
Male:Female		12:11	6:5
SARA	1. Gait (0–8)	2.6 ± 1.8 (1–7)	—
	2. Stance (0–6)	1.7 ± 1.1 (0–4)	—
	3. Sitting (0–4)	1.1 ± 1.2 (0–4)	—
	4. Speech Disturbance (0–6)	1.2 ± 1.2 (0–3)	—
	5. Finger Chase (L) (0–4)	1.3 ± 0.8 (0–2)	—
	5. Finger Chase (R) (0–4)	1. ±–0.7 (0–2)	—
	6. Nose-finger test (L) (0–4)	0. ±–0.8 (0–3)	—
	6. Nose–finger test (R) (0–4)	0.9 ± 0.9 (0–3)	—
	7. Fast alternating hand movements (L)(0–4)	1.2 ± 1 (0–3)	—
	7. Fast alternating hand movements (R)(0–4)	0.9 ± 1 (0–3)	—
	8. Heel-Shin Slide (L) (0–4)	0.8 ± 0.8 (0–2)	—
	8. Heel-Shin Slide (R) (0–4)	0.9 ± 0.7 (0–2)	—
Total SARA (0–40)		11 ± 6.7 (0–27)	—
*Phenotypes	Pure (central) CA	12	—
	CABV	4	—
	CABV + SS	7	—

*Captions:* n: number of participants, CA: Cerebellar Ataxia,

L: Left, R: Right, Data presented as mean ± standard deviation,

CABV: Cerebellar Ataxia with Bilateral Vestibulopathy, SS: Somatosensory.

*Deep phenotyping has not been undertaken in these subjects.

#### Ethics approval and consent to participate

This study was approved by the Human Research and Ethics Committee, Royal Victorian Eye and Ear Hospital, East Melbourne, Australia (HREC Reference Number: 11/994H/16) and supported by the Florey Institute of Neuroscience and Mental Health, Melbourne, Australia through the National Health and Medical Research Council (NHMRC) Grant: GNT1101304 and APP1129595. All the methods in this study were performed in accordance with relevant guidelines and regulations and written consent was obtained from all the participants prior to their enrolment. Informed consent was obtained from both the subjects to publish the images depicted in the Fig. 1.

#### Testing protocol and feature extraction

The objective assessments of the nine neurological tests are grouped into the following 5 domains with a brief description of their execution protocol. A summary of features that proved pivotal in the diagnosis of CA in the related respective study^4,6–11^ has also been included for each test subsection in Table 2.

**Table 2:** Brief description on the STAR characterisation of the 172 features extracted from the 9 neurological tests.

Domain	Sensor	Test	Features	STAR (Ataxic dimensions)	Number of features
Upper limbs (UL)	BioKin	Dysdiadochokinesia (DDK)	Resonant Frequency (RF) of Angle (X, Z) of (LH/RH)	Stability	20
			Magnitude at Resonance (MR) of Angle (X, Z) of (LH/RH)	Stability	
			RF of Angle (Y) of (LH/RH)	Timing	
			MR of Angle (Y) of (LH/RH)	Rhythmicity	
			RF of Acceleration (X, Z) of (LH/RH)	Stability	
			MR of Acceleration (X, Z) of (LH/RH)	Stability	
		Finger Nose (FNT)	RF of Angular acceleration (X, Z) of (LH/RH)	Stability	20
			MR of Angular acceleration (X, Z) of (LH/RH)	Stability	
			RF of Angular acceleration (Y) of (LH/RH)	Timing	
			MR of Angular acceleration (Y) of (LH/RH)	Rhythmicity	
			RF of Acceleration (X, Y, Z) of (LH/RH)	Stability	
			MR of Acceleration (X) of (LH/RH)	Stability	
		Finger Tapping (FIN)	Multiscale entropy (MSE) of Acceleration (X) of (LH/RH)	Stability	20
			Rhythmic Variation (LH/RH)	Rhythmicity	
			Multiscale entropy (MSE) of Acceleration (Z) of (LH/RH)	Rhythmicity	
			MSE of rotational motion (X) of Gyro (LH/RH)	Rhythmicity	
	Kinect	Ballistic tracking (BAL)	Directional change in in H and V axes (LH/RH)	Stability	14
			Kinematic Delay - Index of Performance (LH/RH)	Timing	
			Comprehensive Time Delay in H and V axes (LH/RH)	Timing	
			Dynamic time warping based error in H and V axes (LH/RH)	Accuracy	
Lower limbs (LL)	BioKin	Foot Tapping (FOO)	MSE of rotational motion (Y, Z) of (LL/RL) of Gyro	Stability	14
			MSE of rotational motion (Y, Z) of (LL/RL) of Gyro	Stability	
			Rhythmic Variation (LL/RL)	Rhythmicity	
		Heel-shin (HST)	RF of Acceleration (X, Z) of (LL/RL)	Stability	19
			MR of Acceleration (X, Z) of (LL/RL)	Stability	
			RF of Acceleration (Y) of (LL/RL)	Timing	
			MR of Acceleration (Y) of (LL/RL)	Rhythmicity	
			MR of Angle (Y) of LL	Stability	
Balance	BioKin	Romberg (ROM)	EntropyML (Front/Back & Eyes close/Eyes open)	Stability	14
			EntropyAP (Front/Back & Eyes close/Eyes open)	Stability	
			EntropyAll (Back & Eyes close/Eyes open)	Stability	
			EntropyVT (Front/Back & Eyes close/Eyes open)	Accuracy	
Gait	BioKin	Walking (WAL) (at a fast speed, slow speed and preferred speed)	Fuzzy Entropy-based velocity (Z)	Stability	45
			RF in VT of (LL/RL)	Stability	
			MR in VT of (LL/RL)	Stability	
			Fuzzy Entropy-based velocity (X)	Accuracy	
			RF in ML of (LL/RL)	Accuracy	
			MR in ML of (LL/RL)	Accuracy	
			Fuzzy Entropy-based velocity (Y)	Rhythmicity	
			RF in AP of (LL/RL)	Rhythmicity	
			MR in AP of (LL/RL)	Rhythmicity	
Speech	Condenser microphone	Speech (SPE)	Damping Ratio	Stability	6
			RF	Timing	
			Compensation	Rhythmicity	
			Peak Prominence	Rhythmicity	
			The gap between repeated Ta utterances	Rhythmicity	
			Duration of each Ta utterance	Rhythmicity	

*Captions:* VT = Vertical Axis, AP = Antero-Posterior, ML = Medio-Lateral, RH = Right hand, LH = Left hand,

LL = Left leg, RL = Right leg, H = Horizontal, V = Vertical.

### Speech

- Repeated syllable utterance (SPE): The candidate was required to repeat the consonant-vowel syllable/ta/ for 5 seconds at their preferred speed. The recordings were made using a condenser microphone clipped at an approximate distance of 10 cm from the subject’s mouth in a quiet room with low ambient noise level. An android phone using the program *BioKinMobi* under a professional investigator’s supervision captured the speech. A topographic prominence based automated algorithm was employed to extract six acoustic features from the train of repeated/ta/ syllable utterances^4,6^.
- Regularity of the duration measures the variability in the rhythm of repeated/ta/ (RT) utterance. This is identified as an integral measure of timing deficits extracted from the wave data at 50% prominence.
- Gap regularity measures the time difference variability between two consecutive/ta/ syllable peaks.
- Average peak prominence measured the average relative elevation/peak for a specific/ta/ pulse considered.
- Compensation regularity measured the variability in the differences computed between the peak and its corresponding prominence for a specific/ta/syllabic pulse.
- Damping ratio measured the average of the /ta/ syllables’ damping ratios calculated from the wave data extracted at 75% prominence.
- Resonant frequency measured the average of the /ta/ syllables’ resonant frequency calculated on the wave data extracted at 50% prominence.

### Upper limb

- Rhythmic finger tapping test (FIN): Participants rhythmically tapped their index finger against a horizontal surface (e.g. table top) at their preferred speed and duration. A *BioKin* was mounted on the dorsum of the pointing index finger for data acquisition. The first 3 Principal Components (PC) of multiscale entropy measured from the X and Z axes of accelerometer signals and X axis of gyroscope signals and the coefficient of variation of the inter-tap interval measuring the irregularity of the rhythm were the selected features^7^.
- Finger-nose test (FNT): Participants were required to touch their nose with their pointed index finger and then, using the same finger, reach out and touch the clinician’s finger placed approximately 25 cm from the subject’s nose. Hand movements were measured by a *BioKin* attached to the dorsum of the hand of the pointing index finger. Resonance frequency and amplitude at resonance frequency were the critical features^8^ as captured by the *BioKin* attached to the palm of the pointing index finger. The frequency domain description of acceleration and angular velocity was used to capture the resonance in each orthogonal axis (X, Y and Z)^8^.
- Dysdiadochokinesia test (DDK): Participants were required to place the dorsum of one hand on the palm of the other hand, as depicted in Fig. 1. The participants were then instructed to pronate their hand, so that palm side faces downwards to rest on the palm of the other hand. The subject is also instructed to pronate and supinate alterntely between these two positions as fast and precise as possible. The rate of alternation is extracted from the *BioKin*’s IMU attached to the wrist. This test examined for inability to co-ordinate movement. The rate of alteration of pronate and supinate, resonance frequency and amplitude at resonance frequency were the critical features^8^.
- Ballistic tracking (BAL): Participants were required to point to the target on a monitor screen. The movement of the pointing index finger was detected using the Kinect camera and was presented as a marker on the screen. The objective is to accurately follow the target via the projected (with the Kinect camera) marker on the screen when the target is moving rapidly and randomly from point to point on the monitor. The following extracted features displayed a significant level of correlation with the disability level captured by the standard clinical measure SARA^9^:

- Error: The distance between marker and target trajectories, measured using Dynamic Time Warping method in the Horizontal(H) and Vertical(V) axis.
- Comprehensive time delay: This was calculated as the cross-correlation for the two-time sequence, marker and target.
- Kinematic delay: This was obtained using the index of performance measurement in Fitts’ law. The feature is to measure the performance of the subject in reaching a target position.
- Directional Change in H and V axis: This is the number of times the participant altered their acceleration which was measured in terms of directional change. This feature contained information of over/undershooting as well as the performance of the subject during the test. Higher level of dysmetria inferred a greater error rate as per the difference between the target and the marker trajectories.

### Lower limb

- Heel-shin test (HST): Participants were required to place a heel on the opposite knee and run it along the tibia, between the heel and the knee repetitively and as accurately as possible. The *BioKin* was attached to the dorsum of the foot. Resonance frequency and amplitude at resonance frequency were the critical features^8^.
- Rhythmic foot tapping (FOO): Participants were required to rhythmically tap each foot against a horizontal surface (e.g. floor). The first 3 Principal Components (PC) of multiscale entropy measured from the X and Z axes of accelerometer signals and X axis of gyroscope signals, and the coefficient of variation of inter-tap interval measuring the irregularity of rhythm were the selected features^7^.

### Balance

- Romberg test (ROM): Participants were required to stand with feet together then with feet apart, arms by the sides for as long as possible (up to 30 seconds); first with eyes open and then with eyes closed. One *BioKin* was positioned approximately on the xiphisternum by means of an elastic neoprene belt. The second *BioKin* was attached on the upper-back location, in the mid-line just below the neck. Fuzzy entropy technique was employed on the postural sway velocity deduced from the measured truncal accelerations. The entropy values^23^ of the deduced velocity was considered primarily as a measure of neural motor control during a quiet standing posture of which a significant portion is proportional to body sway velocity. Uncertainty in the velocity measurement contained a significant level of information with respect to truncal instability^10^.

### Gait

- Gait test (WAL): Participants were required to walk for 5 meters and return which was repeated 10 times. The subject’s movements were captured by the built-in inertial sensors of a smartphone attached at the xiphisternum by means of an elastic neoprene belt and two *BioKin* sensors, attached to each ankle. The sensor was positioned so that its X, Y and Z axes captured ML (Medio-Lateral), AP (Antero-Posterior) and VT (Vertical Axis) movements respectively. In each orthogonal axis (X, Y and Z), the frequency domain description was used to capture the resonance^11^. For each subject, the magnitude and the resonance were used in each axis to form a feature vector. Another feature, fuzzy entropy-based velocity irregularity measure for truncal abnormality (VI) was chosen in the study^11^ to measure the gait randomness or uncertainty level during walking. The study in^23^ introduced fuzzy entropy (FuzzyEn) to capture truncal ataxia.

In reference to the ataxic cohort enrolled in our study, for those with a SARA score 7 for walking – in the instance a gait aid is required, the patient is requested to perform the test with the use of the appropriate gait aid (*i.e*. a single point stick or Four-wheeled frame (4WF)).

#### Ataxic dimensions (STAR)

The works of Gordon Holmes are often cited as having a foundational influence on our understanding of the clinical symptoms and signs of cerebellar lesions^2,3,24^. In our study, we revisited Holmes’ approach of characterising the movement of subject’s with cerebellar dysfunction in terms of four dimensions (Stability, Timing, Accuracy & Rhythmicity).

- Stability **(S)**: This relates to stability in the platform (of execution). The platform is the joints and muscles that are relatively fixed and allow the moving body part to execute a task accurately. For example, the DDK task requires relative stability of the shoulder and elbow flexion and extension for efficient execution. Relative instability results in an increase in unnecessary movements in secondary axes.
- Timing **(T)**: When CA is present, tasks that have a time constraint, such as BAL usually are found to have increased latency before the movement begins and the task is executed at a slower speed, because a less direct course is taken. The same features are often apparent even in the absence of time constraints. These features are more apparent when the CA is more severe, suggesting that, subconsciously, timing is a neutral trade off to complete the task. In computational terms, we recognised it as the error between the goal against what is achieved, likely to be impacted by the following two:

- Time for the subject to initiate a moment.
- Time to complete a movement (speed).
  - Accuracy **(A)**: Conceptually, a task might be completed slowly but follow the most efficient target. Under these circumstances we will consider this to be an “accurate” performance. When a less direct path is followed (for instance in the BAL task) or there is under or over shoot, then the task will be ‘errors’ compared with a control performance (acknowledging that this may also be associated with timing errors). In computational terms, in this study we recognise it as error between the goal/space objectives against what is achieved in a spatial context (static).
  - Rhythmicity **(R)**: Irregularity in repeated movements.

The features for each test in our proposed COA System are assigned to the aforementioned dimensions through the following 2-step approach:

(a) The execution axis is the direction of the primary movement required to execute the intended task and would attribute to rhythmicity or timing dimension.
  (b) Any deviation from the most efficient or the standard path required to execute the task would be considered as accuracy features.
Excessive movements in the other axes would be considered as secondary movements and attribute to the stability dimension.

**Figure 2:**
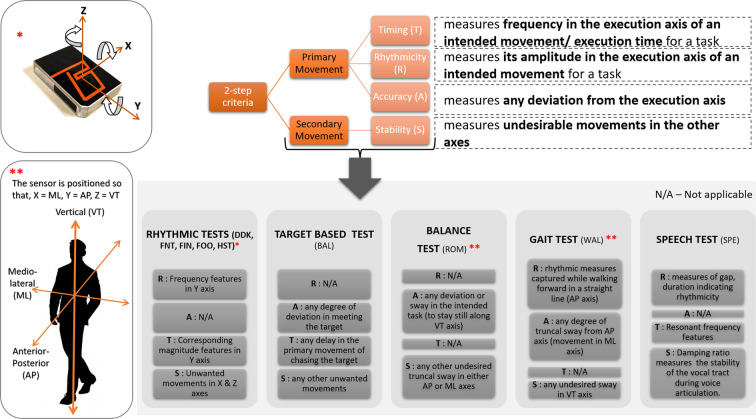
STAR Labelling Criteria.

A pictorial representation in Fig. 2 illustrates the STAR interpretation for each domain, as per the proposed 2-step approach.

In *repetitive tests* (DDK, FNT, FIN, FOO, HST), resonance frequency along the y-axis (primary) contributes to speed and hence is considered as a timing feature whereas the magnitude of resonance is considered as a rhythmic feature. The secondary movements/disturbance present in other axes are termed as stability features.

For *target based tests* (BAL), any delay in the primary movement of chasing the target is considered as a timing feature; how well a target is met or any degree of deviation in meeting the target defines the performance of the participant and hence measures accuracy; any other feature catering to excessive/inefficient movements are marked under stability.

The *gait test* consists of walking forward in a straight line (along the AP axis) at a regular pace by lifting and setting down each foot in a rhythmic fashion. This would infer rhythmicity information whereas the extent of truncal sway from AP axis (that is, movement in ML axis) will infer accuracy information. Moreover, any undesired sway in VT axis are considered as stability feature.

For *balance test*, a participant is expected to maintain a steady straight posture along their VT axis. This being their primary movement, any deviation or sway in VT axis will account for inaccuracy and any other undesired truncal sway in either AP or ML are considered as stability features.

For *speech test*, the features measuring the rhythmic nature of the repeated /ta/ utterances, for example, gap between consecutive /ta/ utterances, duration of a /ta/ are considered as rhythmicity features and the resonant frequency feature as a timing feature. Lower damping ratio indicates a higher oscillation. Hence, the lower damping ratio of a /ta/ utterance, as an ataxic acoustic feature, indicates instability of the vocal tract during voice articulation.

A summary of the nine tests in 5 domains, generating 172 features is presented with their STAR interpretation in Table 2.

#### Clinical assessment

CA was scored by an experienced clinician according to the SARA scale while subjects with ataxia performed each task. SARA is a clinical scale developed by Schmitz-Hübsch *et al*.^25,26^ which assesses a range of different impairments in cerebellar ataxia, ranging from speech to balance. The scale is made up of 8 categories with scores ranging as, gait (0–8 points), stance (0–6 points), sitting (0–4 points), speech disturbance (0–6 points), finger chase (0–4 points), nose-finger test (0–4 points), fast alternating hand movement (0–4 points), heel-shin slide (0–4 points). Once the clinician assesses each of the 8 categories for an individual, they can further compute the cumulative score ranging from 0 (no ataxia) to 40 (most severe ataxia) to determine the ataxic subject’s severity of ataxia. In our study, to avoid any subjective bias, one clinician assessed all the tasks.

#### 3-tier evaluation scheme of COA system

**Figure 3:**
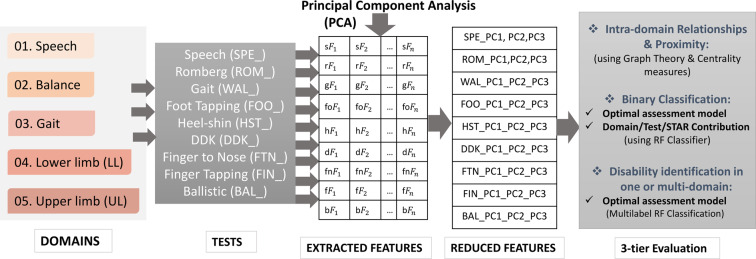
3-tier Evaluation process flowchart of COA System.

The techniques to be incorporated in the proposed instrumented system (COA system) are demonstrated through a flowchart (Fig. 3) and outlined in the following subsections.

### Feature reduction and statistical analysis

Original feature extraction for each test were based on relevant previous studies^4,6–11^. A total of 172 features were identified as critical for objective assessment of individual tests. Process measurements contain many correlated or redundant data. It is important to remove them and extract the features that carry the most independent relevance. Principal Component Analysis (PCA)^27^ is a data compression, extraction and visualisation tool used to transform several associated factors into a group of uncorrelated variables. PCA is used to compress the original 172 features into 27 PCs (3 PCs from each of the 9 tests) (Fig. 3). Moreover, critical information does not come from a single variable of an individual test, but frequently stems from the relationship between variables, i.e. how they co-vary. PCA is the most appropriate among the commonly used multivariate statistical methods for evaluating such information because it can manage big numbers of highly correlated, noisy and redundant factors.

The p-value for hypothesis testing is calculated for the distributions of the resulting 3 PCs (PCs 1-2-3) of each test with respect to each of the 5 domains (Speech, upper limb, lower limb, gait, balance) to determine if the groups of subjects (control and ataxic) differ significantly. In each domain, individuals with SARA measures greater than zero, are grouped as ataxic, and controls and subjects who scored a SARA score of 0 for a particular test are grouped as normal. Non-parametric statistical tests (Kolmogorovâ€“Smirnov (KS) and Mann-Whitney-Wilcoxon (MWW)) are adopted to avoid assumptions on data distribution.

### Graph theory & centrality measures

#### Visual quantification of the test/domain dependencies

Graph theory is applied to obtain further insights into the relationships among tests and domains. Tests and domains were assigned to the nodes of a network, which joined up the nodes by edges with lengths representing Spearman’s rank correlation coefficients (*ρ*). The centrality of a node indicates the number of edges adjoining that node and the proximity to all other nodes which is considered as an indication of the node’s importance. The frequency that a node appears on the shortest path between two other nodes is also a measure of importance. The Minimum Spanning Tree (MST) analysis^28^ is used in our study as a reliable measure for comparing the networks across different groups since it is unbiased and does not require arbitrary parameter settings^29^. MST has only been recently applied to brain networks^29^ and identification of critical genes in diabetes mellitus^30^. The MST is a sub-graph that connects all nodes to reduce the total edge length. In this sense, the MST is the “backbone” network that encapsulates the inter-test/domain dependencies. To measure proximity, we use the following mapping to translate the rank correlation coefficients of Spearman (*ρ*) into distances.

1 $$f(\rho )=1-\rho ,$$

2 $$or,f(\rho )=\sqrt{2(1-\rho )}.$$

MST of this graph is computed by assigning the tests/domains to the nodes of a network and joining the nodes via edges with lengths given by *ρ*.

#### Overview of test/domain importance using graph centrality measures

Representing the tests and domains in a graph form enables the quantification of the relationships between them. Since mathematical graphs intrinsically characterize node significance measurements, the tests/domains assigned to those nodes are considered highly relevant in measuring CA. Feature importance score is then computed on the entire graph using popular Centrality Measures like Degree Centrality, Closeness Centrality and Betweenness^30^. The Incidence or Degree Centrality of a node in a given graph counts the number of edges adjoining that node which is mathematically defined as,

3 $${C}_{D}(N)={\rm{\deg }}(N),$$

where, *g* := (*N*, *e*) is the given graph with |*N*| nodes and |*e*| edges. In a connected graph, the average length of the shortest path between the node and all other nodes in the network is denoted as the normalized Closeness Centrality (or Closeness) of a node. Therefore, a high value of Closeness implies that the node is central or significant. Closeness is defined as the reciprocal of the sum of the distances from the node to all other nodes, that is,

4 $${C}_{C}({N}_{1})=\frac{1}{{\sum }_{y}d({N}_{2},{N}_{1})},$$

where *d*(*N*_1_, *N*_2_) is the distance between vertices *N*_1_ and *N*_2_. Likewise, a node’s Betweenness calculates how frequently that node appears between two other nodes in the graph on the shortest path. A high value of Betweenness means the node is relevant. The Betweenness of a node *N* is denoted as,

5 $${C}_{B}(N\rangle =\sum _{{N}_{1}\ne N\ne {N}_{2}\in N}\,\frac{{\sigma }_{{N}_{1}{N}_{2}}(N\rangle }{{\sigma }_{{N}_{1}{N}_{2}}},$$

where ${\sigma }_{{N}_{1}{N}_{2}}$ is the total number of shortest paths from node *N*_1_ to node *N*_2_ and ${\sigma }_{{N}_{1}{N}_{2}}(N)$ is the number of those paths that pass through *N*.

### Classification experiment

#### Binary classification

The next step of the scheme consists of a diagnosis or a binary classification problem comparing the discrimination performance of each of the individual tests, the combined 9 test and two reduced subsets using a Random Forest (RF) classifier^31^. Each feature contributed to each one of the 4 Holmeshian dimensions to varying degrees (the weights) which were computed accordingly for the overall 9 test as well as for the optimal subset of tests.

#### Multilabel classification

In our study, a Random forest based adopted algorithm for Multilabel Classification^32^ is used.

The feature input in the multilabel classification problem of our study consisted of 27 principal components from all tests (3 PCs x 9 tests). The Target was to identify the disabilities in 5 domains (0: normal; 1: ataxic). For example, a participant is represented by the domains of speech, upper limb, lower limb, gait and balance; and the possible label powerset representation of this is a multi-class classification problem with the classes [0 0 0 0 0], [1 0 0 0 0], [0 1 0 0 0], [0 0 1 0 0], [0 0 0 1 0], [0 0 0 0 1], [1 1 0 0 0], [1 0 1 0 0], …, [1 1 1 1 1] where, for example, [1 0 1 0 0] denotes a participant whose domains of speech and lower limb are affected whereas the domains upper limb, gait and balance are unaffected.

#### Feature importance (or rank) in RF model

At the very outset, the optimal leaf size in an RF classifier is verified by comparing Mean Squared Errors (MSE) obtained by classification for various leaf sizes (5, 10, 20, 50, and 100). The optimal leaf size should yield the lowest MSE values. Once we have estimated the optimal leaf size, a larger ensemble is grown and used to estimate feature importance. To compute the feature importance in the Random forest diagnostic model, initially, the MSE of the model with the original variables is calculated. Then, the values of a single column (representing feature 1 for n observations) are permuted and the MSE is calculated again. For instance, if a column takes the feature values *x*_1,_ *x*_2,_ *x*_3,_ *x*_4_ and a random permutation of the values results in *x*_4,_ *x*_3,_ *x*_1,_ *x*_2_; then this will result in a new MSE. The difference in MSE is averaged over all trees in the ensemble and divided by the standard deviation taken over the trees for each variable. The greater this value, the more significant the variable is. The difference is expected to be positive, but if it is a negative number, then it implies that the random permutation worked better inferring that the feature does not have a role in the prediction and is not deemed important.

#### STAR computation

Once the importance/rank of the 3 PC features is evaluated for a specific test through the Random Forest ranking scheme, the weight of the original feature is computed as follows:

6 $$feature\_weights=WOF\_InPC{1}^{\ast }R\_PC1+WOF\_InPC{2}^{\ast }R\_PC2+WOF\_InPC{3}^{\ast }R\_PC3,$$

where WOF: Weight of this feature in a PC component; R: Rank of the PC feature in RF model. Since each feature relates to one of the 4 Holmeshian dimensions, the contribution of the overall Stability, Timing, Accuracy and Rhythmicity dimension is the accumulated weigtage of all the Stability, Timing, Accuracy and Rhythmicity features respectively.

#### Cross validation (CV)

For both the classification problems, the data is stratified using a Leave-one-out (LOO) CV technique. Cross-validation in multilabel settings is complicated by the fact that the ordinary (binary/multiclass) way of stratified sampling is not applicable; alternative ways of approximate stratified sampling have been suggested in^33^. So, in our study, the multi-label stratification was performed using an iterative technique.

#### Evaluation metrics

The performance of the classifier is evaluated using the metrics, Precision, Recall, F1 score, Accuracy and Matthews Correlation Coefficient (MCC)^34^. These metrics are calculated for each domain based on the predicted values after each validation in LOO (34 times). General precision, recall, F1 score, Accuracy of multilabel classification problem are the average of the results through LOO in the 5 domains. For example,

7 $$general\_precision=sum(precision\_values\_in\_5\_domains)/5.$$

Feature ranking through a RF train & validation with LOO is the average of all the rank in each training and validation phase, for both the binary and multilabel classification problems.

## Results and Discussion

The experimental results of applying all incorporated methods in the proposed instrumented system (COA system) for the prediction of CA are explained and discussed in this section.

### Projected PC feature distribution in 5 domains - statistical analysis

The Principal Components (PCs 1-2-3) for all the tests were investigated to fulfill the normality distribution assumption using the Kolmogorovâ€“Smirnov test. It was followed by hypothesis testing to examine the group differences for normal and ataxic groups with respect to the 5 domains using MWW test. For each test, there were statistically significant differences between at least one PC of normal and ataxic subjects at 5% significance level (bold indicating significant p values in Table 3). In addition, box plots were also presented (see Supplementary Fig. S1) to demonstrate the distribution of the PCs 1-2-3 with respect to the 5 domains. The PCs, FNT_PC1, FIN_PC3, ROM_PC1, BAL_PC1 were statistically significant in differentiating the normal and CA groups in all the 5 domains with p < 0.05 (rows indicated in bold in Table 3). Significant differences between ataxic and normal groups are depicted in Table 3; p showing the statistical difference between normal and ataxic groups with respect to the 5 domains with the significant p values (p < 0.05) are highlighted.

**Table 3:** Significant difference between ataxic and normal groups; p denotes the statistical difference between ataxic and normal groups with significant p values (p < 0.05) are highlighted.

Parameters	Mean Standard Deviation	p-value	Speech	Upper limb	Lower limb	Balance	Gait
**DDK_PC1**	−3.08	20.53	0.5609	0.7049	0.8476	0.5102	0.4774
**DDK_PC2**	6.07	18.53	0.0346	**0.0044**	0.0450	**0.0045**	**0.0004**
**DDK_PC3**	1.66	8.56	0.4135	0.7298	0.6489	0.6331	0.6356
**FNT_PC1**	2.48	6.67	**0.0011**	**0.0037**	**0.0019**	**0.0029**	**0.0005**
**FNT_PC2**	−0.05	3.51	0.2746	0.1640	**0.0186**	0.0727	0.3020
**FNT_PC3**	−0.10	1.95	0.1413	0.2596	0.3593	0.2651	0.3638
**FIN_PC1**	0.13	1.42	0.1597	**0.0082**	0.0907	**0.0039**	**0.0003**
**FIN_PC2**	0.09	0.48	0.6280	0.6146	0.6594	0.7431	0.8650
**FIN_PC3**	−0.11	0.27	**0.0104**	**0.0147**	**0.0244**	**0.0001**	**0.0007**
**FOO_PC1**	−0.05	0.36	0.7823	0.9709	0.8996	0.2290	0.3600
**FOO_PC2**	−0.01	0.12	0.1744	0.1223	**0.0155**	**0.0401**	**0.0694**
**FOO_PC3**	−0.03	0.11	0.2111	0.1271	0.4476	0.1633	0.2046
**HST_PC1**	−0.23	2.21	0.0788	0.0211	0.0980	**0.0033**	**0.0002**
**HST_PC2**	0.09	1.60	0.2627	**0.0007**	**0.0304**	0.1262	**0.0043**
**HST_PC3**	−0.14	1.35	0.4452	0.2329	0.7194	0.8238	0.6458
**ROM_PC1**	−0.04	0.90	**0.0002**	**0.0000**	**0.0000**	**0.0001**	**0.0000**
**ROM_PC2**	−0.04	0.50	0.3212	0.7304	0.8556	0.7555	0.9044
**ROM_PC3**	0.05	0.54	0.0847	0.9369	0.9956	0.6591	0.8574
**SPE_PC1**	7.30	28.78	0.6199	0.7124	0.8723	0.8307	0.7705
**SPE_PC2**	0.06	0.14	0.4834	0.2610	0.4275	0.0641	0.1144
**SPE_PC3**	−0.01	0.06	**0.0481**	**0.0362**	0.0429	0.1071	0.2245
**WAL_PC1**	0.10	39.77	**0.0245**	**0.0474**	0.1933	**0.0201**	**0.0062**
**WAL_PC2**	4.48	25.45	0.8141	0.7959	0.5848	0.7908	0.7117
**WAL_PC3**	−0.39	19.28	0.3604	0.7329	0.6273	0.6789	0.5147
**BAL_PC1**	−0.79	2.64	**0.0014**	**0.0117**	**0.0064**	**0.0014**	**0.0014**
**BAL_PC2**	0.15	1.75	0.3897	0.6231	0.9053	0.4121	0.4136
**BAL_PC3**	−0.14	0.99	0.4886	**0.0521**	0.1494	0.0891	0.2088

Recent publications^35,36^ on current global epidemiological scenarios of ataxia estimate an overall ataxia occurrence rate of 26/100,000 in children and an occurrence rate of 2.7/100,000 for dominant hereditary cerebellar ataxia. These studies estimate the frequency of recessive hereditary cerebellar ataxia as 3.3/100,000. In our study, for a given large effect size (Cohen’s d of 2.384), we determined a minimum sample size of 34 (Controls = 11, CA subjects = 23) by power analysis, with the error probability (*α*) set at 0.05 and a false negative rate (*β*) set at 0.1 (that is a power of 0.9).

### Intra-domain relationships and proximity - graph theory & centrality measures

**Figure 4:**
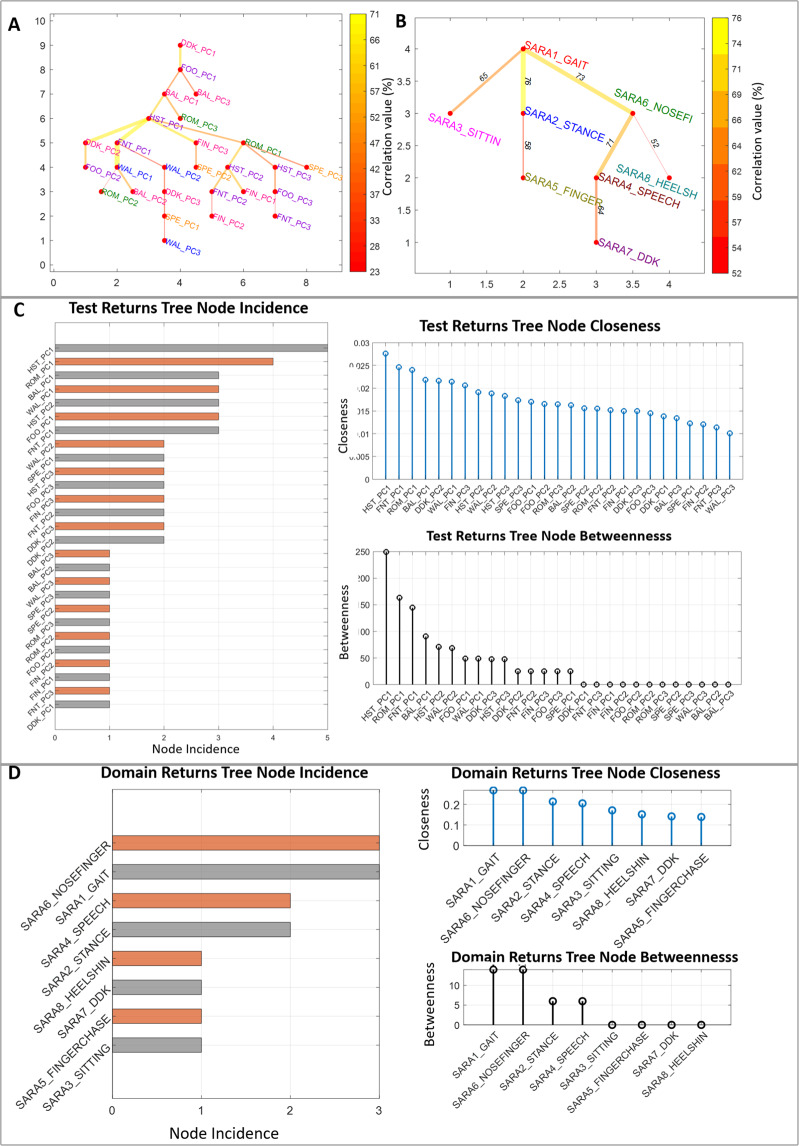
Minimum Spanning Tree (MST) of test w.r.t to the PC features belonging to (**A**) the 9 tests and (**B**) the 8 SARA tests. Centrality measures *viz*., Incidence, Closeness and Betweenness computed from the MSTs w.r.t to the PC features belonging to (**C**) the 9 tests and (**D**) the 8 SARA tests. SARA tests for gait, stance, sitting, speech disturbance, finger chase, nose-finger, fast alternating hand movement and heel-shin slide are labelled as SARA1_GAIT, SARA2_STANCE, SARA3_SITTIN, SARA4_SPEECH, SARA5_FINGER, SARA6_NOSEFINGER, SARA7_DKK, SARA8_HEELSH respectively. SARA5_FINGER, SARA6_NOSEFINGER, SARA7_DKK, SARA8_HEELSH are the respective mean values for the bilateral SARA assessments for the motor activities of the four extremities (items 5–8).

The MST of the 27 PC features of all tests shows that Upper limb peripheral tests and Gait have a strong correlation (0.77) (Fig. 4A) and agrees with SARA ratings (Fig. 4B). However, different tests were important for this correlation in the instrumented version (HST, ROM, FNT & BAL) and the clinical version (SARA6_NOSEFINGER, SARA1_GAIT, SARA2_STANCE & SARA4_SPEECH). The results of the three Centrality Measures computed from the respective MSTs are highly correlated (*ρ* > 0.95) (see Supplementary Fig. S2) and the test rank order based on our feature (Fig. 4C) are similar to those obtained from SARA assessment test scores (Fig. 4D).

To obtain the MST of the SARA ratings, the mean values were calculated for the bilateral SARA assessments for the motor activities of the four extremities (items 5–8).

### Classification experiment

#### Binary classification comparison and optimal subset selection (subset 2)

**Figure 5:**
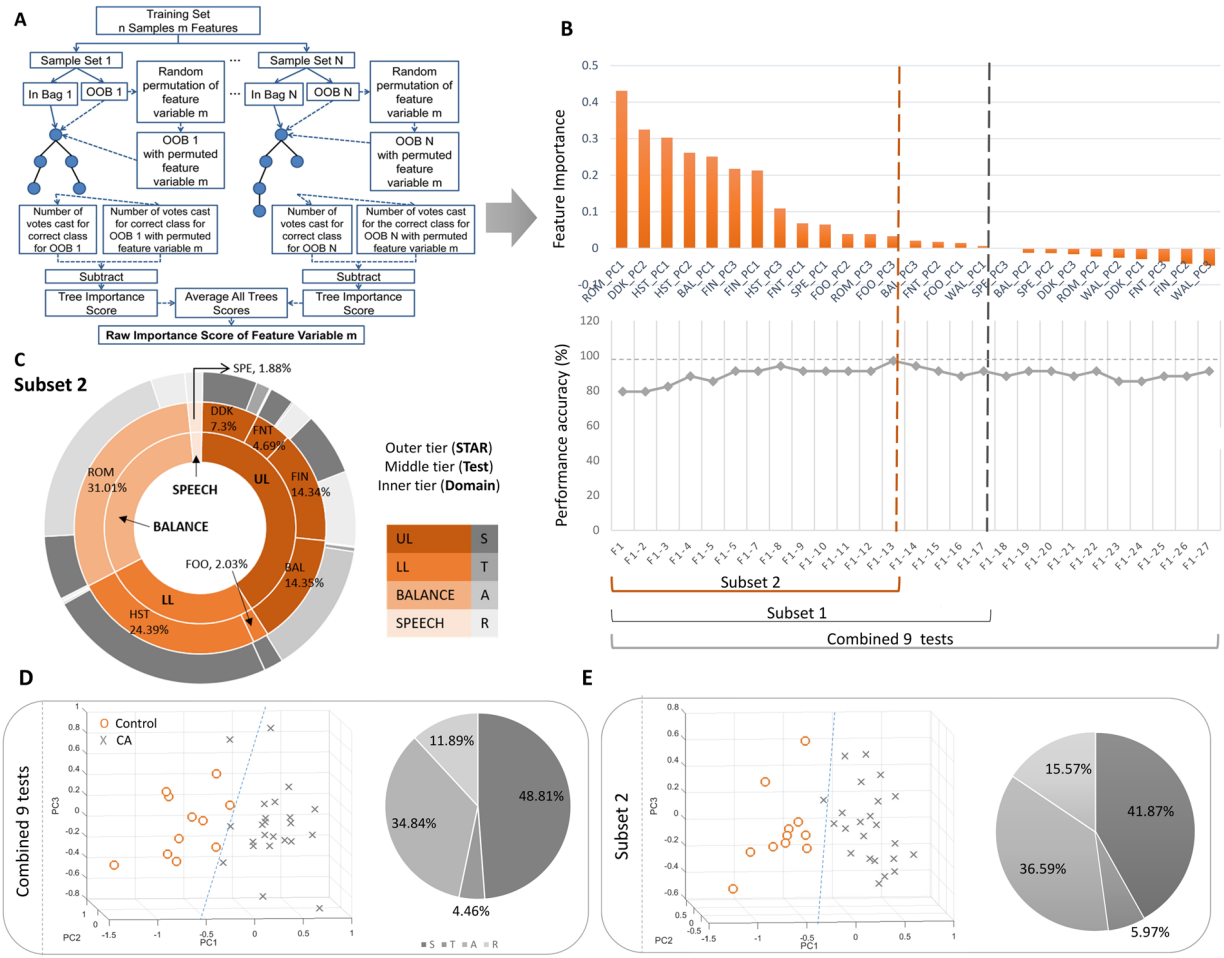
Binary classification, (**A**) Feature Importance calculation flowchart for combined 9 tests, (**B**) Selection of optimal number of test PCs using Random Forest, (**C**) Test, Domain and STAR distribution from Subset 2, (**D**) Scatterplot of combined 9 tests and the corresponding STAR distribution, (**E**) Scatterplot of Subset 2 and the corresponding STAR distribution.

Table 4 compares the performance of the CA diagnosis classification of individual tests, the combined 9 tests and two reduced feature subset using Random Forest. The combined 9 tests demonstrated a performance accuracy of 91.17% (F1 score = 84.21%, Precision = 72.73%, Recall = 100%), which was higher than any of the individual 9 tests. This is a greater number of tests than in our previous studies^4–11^ and in the available literature^15,16^ and provides a comprehensive overview of CA. Figure 5B illustrates the feature importance of each input feature through bar plots, as computed through the process illustrated in Fig. 5A. The blue bars represent PC features with negative feature importance. Subset 1 (with 17 PC features) was obtained after removing those PC features whose feature importance in the RF classifier model for the combined 9 tests was negative (Fig. 5B). We modelled another subset (Subset 2) and continued to add the PC features one by one in the decreasing order of their feature importance until there was no further improvement in the discrimination accuracy. This is the optimal subset (with 13 top PC features). It demonstrated the highest performance accuracy of 97.06% (F1 score = 95.24%, Precision = 90.91%, Recall = 97.06%).

**Table 4:** Diagnosis Performance Comparison using a Random Forest Classifier.

Test	Precision(%)	Recall(%)	F1 Score(%)	Accuracy(%)	MCC
DDK (DDK_)	54.55	66.67	60	76.47	0.44
Finger to Nose (FNT_)	45.45	38.46	41.67	58.82	0.1027
Finger Tapping (FIN_)	54.55	75	63.16	79.41	0.5057
Ballistic (BAL_)	54.55	54.55	54.55	70.59	0.3281
Foot Tapping (FOO_)	18.18	33.33	23.53	63.33	0.0097
Heel-shin (HST_)	81.82	90	85.71	89.2	0.7954
Romberg (ROM_)	72.73	72.73	72.73	82.85	0.5968
Speech (SPE_)	45.45	55.56	50	70.56	0.2976
Gait (WAL_)	18.18	18.18	18.18	47.06	−0.2095
Combined 9 tests	**72.73**	**100**	**84.21**	**91.17**	**0.8021**
Subset 1 (top 17 features)	72.73	100	84.21	91.17	0.8021
Subset 2 (top 13 features)	**90.91**	**100**	**95.24**	**97.06**	**0.9334**

Interestingly, the Gait test (WAL) did not contribute in improving the performance accuracy of our system and were excluded from Subset 2. This gave the confidence on the inference made on the test/domain ranking based on Centrality Measures. The high correlation observed between the Upper limb peripheral tests and Gait test in the MST elucidates the fact why WAL test features do not contribute to the discrimination and are redundant in the optimal Subset 2. At least 1 PC feature belonging to the other 8 tests contributed in improving the accuracy of the model to varying proportions and were included in the 13 features of Subset 2. Compared with Fig. 5D (based on the combined 9 tests), the CA versus control group distribution in Fig. 5E (based on the Subset 2) is much more distinctiv. Also,, 5D has a better separation in the scatter plot (smaller intra-cluster distance and larger inter-cluster distance), as supported by its higher value of accuracy (97.06%).

#### Domain, test and star contribution from subset 2

The STAR dimension contribution in the optimal Subset 2 as depicted in the Fig. 5D is listed as, 41.87%, 5.97%, 36.59 and 15.57% of Stability, Timing, Accuracy and Rhythmicity respectively. The contribution of ataxic dimensions are in the same order (S, A, R, T) for both the combined 9 tests and the Subset 2 (Fig. 5D,E). This confirms the fact that exclusion of WAL test features (and its corresponding Stability, Accuracy and Rhythmicity features) in Subset 2 did not affect the STAR distribution. The findings from this section also highlighted the fact that the features contributing significantly to the correlation with clinical assessments were not always recognised as clinically important (for example, stability features were deemed prominent in the data set required to model dysdiadochokinesia although it is not recognised as important by clinicians). The highest contribution in diagnosing CA is from the domain Balance and ROM test (31.1%). This is a new finding that is not in line with the general clinical conventions. PC feature, ROM-PC1 and hence, the pre-engineered feature, entropy of the ROM test with eyes closed captured from the sensor attached to upper back in the Vertical axis has the maximum contribution. Other significant contributions come from the peripheral tests in the descending order: HST > BAL > FIN > DDK > FNT (Fig. 5C).

#### Optimal subset selection in multilabel classification

To identify how the PCs of a specific test are mapped to the disability of the 5 domains (target) in CA based on the selected features by us, we investigated the underlying multilabel classification problem through four popular algorithms; Random Forest, Multi-layer Perceptron (MLP), K- Nearest Neighbour (KNN) and Decision Tree (DT)(Table 5). Random Forest performed best with an overall multilabel classification accuracy of 82% (Precision = 83.3%, Recall = 85.6%, F1 score = 84.3%), followed by 77.3% in Decision Tree (Precision = 80.6%, Recall = 74.6%, F1 score = 76.9%).

**Table 5:** Multilabel Classification comparison with different classifiers.

Algorithms	Precision(%)	Recall(%)	F1 score (%)	Accuracy
**Random Forest (RF)**	**83.3**	**85.6**	**84.3**	**82**
**Multi-Layer Perceptron (MLP)**	75.4	73.8	74.5	72.1
**K-Nearesr Neighbour (KNN)**	64.2	65.4	64.6	59.3
**Decision Tree (DT)**	80.6	74.6	76.9	77.3

**Figure 6:**
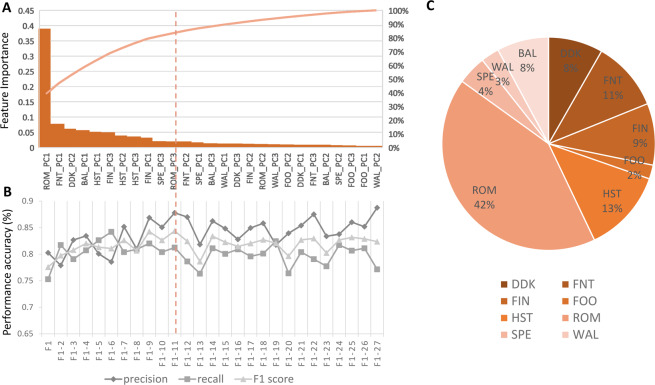
Multi-label classification, (**A**) Feature Importance calculation flowchart for combined 9 tests, (**B**) Selection of optimal number of test PCs using Random Forest, (**C**) Test contribution in the multi-label classification.

It is evident that the highest contribution in mapping a specific test to the disability in 5 domains of CA is from ROM (42%) test. The PC feature, ROM-PC1 and hence the selected feature, entropy of the ROM test with eyes closed captured from the sensor attached to upper back in the vertical axis incurred the maximum contribution. Other significant contributions came from peripheral tests in the descending order: FNT > HST > FIN > DDK > BAL (Fig. 6C). Based on the performance metric F1 score, we further used the Random forest classifier to determine an optimal subset. We selected a subset of the top 11 features resulting a comparable F1 score (84.3%) in accordance to the law of parsimony (Fig. 6A,B).

Fusing data from disparate sources (IMU and Kinect camera) enabled the precise tracking of the limb joint movements using optical and orientation information. This improved the reliability of the proposed system and compensated for any inaccuracies of one sensor in segregating the features into the four dimensions.

From a clinical perspective, the proposed instrumented COA system can help clinicians to function as a tool to support the diagnosis of CA and provide an explanation for informed decision-making. Our findings paved the way to enhance the utility of objective measures for clinical assessments. In addition, the benefits obtained through the incorporation of multimodal sensors can be combined into a combination of three basic aspects; reduced cost, reduced time and additional information. A well-designed multimodal interface that fuses different types of sensors allows additional features in the overall movement to be identified.

Demographic factors, including age of onset, age of diagnosis and other environmental factors do not influence age and gender-related to ataxia^20–22^. We focused here on ambulatory subjects because non-ambulatory subjects provide a further level of complexity. Additionally, this study utilised subjects who were able to complete all the tests listed so that we can support proof of concept in the distillation and combination of key-instrumented metrics – the STAR concept. However, we aim to address this in a future study where this cohort will be recruited and tested as per a modified suite (standing test cannot be performed in non-ambulant subjects) of instrumented testing.

The SARA was chosen because it is more widely used than other scales such as the CCFS. the SARA has been shown to be a reliable and valid measure of CA for upper limb, lower limb and gait function and has at least 8 clinical trials evaluating its use in ataxia cohorts^37^. Our aim was not to model the SARA or the CCFS with the aim of producing an instrumented version of either of these scales. We required one of the clinical CA scales in the first instance to ensure that we were able to detect the abnormalities that such clinical scales are able to identify, and to then move beyond this in developing instrumented devices which can identify very early signs of CA, and also to be able to grade the severity of an individual’s impairment.

The data set is a limitation in this study owing to certain factors. In general, ataxias as a whole are rare. They involve multitude of genetic factors coupled with variable disease progressional rate. Hence, appropriate diagnosis and distinguishing them from other neurodegenerative diseases poses a big challenge. Validation of the proposed system in non-clinical settings in a wider cohort would enhance its value and render it fit for inclusion into routine clinical practice.

## Conclusion

To measure clinical progression in CA requires the ability to measure established ataxia with less variability than is currently the case. It is the variability that extends the time for statistically significant change to occur. Neurologists do not consistently agree on the severity of ataxic signs, and this is a major motivation in our work and that of others. In this study, the focus was on ambulatory subjects because non-ambulatory subjects provide a further level of complexity. To the best of our knowledge, this is the first comprehensive approach to determine an optimal, easy to use instrumented system in CA diagnosis covering all the 5 domains (*viz*., speech, upper limb, lower limb, gait and balance) and unveils the intra-domain relationships. A reduced subset with 13 PC features ranked according to feature importance demonstrated better performance accuracy of 97.06% (F1 score = 95.2%) as compared to the individual tests and combined 9 tests in discriminating CA/controls. A Random Forest binary classifier with LOO validation scheme was used. Gait (WAL) test did not contribute to this discrimination significantly whereas balance (ROM) test contributed the highest (31.1%). A labelling criterion is introduced in this study to characterise the dominant features in each test into Holmesian dimensions (STAR). Importance of each test/domain was calculated with centrality measures using our COA scheme and compared with SARA. The MST showed that Upper limb peripheral tests and Gait have a strong correlation (0.77), based on our features and it agreed with the SARA rating. The mapping between the 27 PCs deduced from the features extracted from the objective assessment of 9 tests and the 5 domains were identified using Random Forest approach by transforming this scenario into a multi-class classification problem. The highest contribution in this mutilabel classification was from ROM (42%), followed by the peripheral tests. A Random Forest classifier achieved the highest F-score (84%) with the combined 9 test features. A reduced feature subset consisting of top 9 features with comparable F-score performance was selected according to the parsimony principle. These findings demonstrate the potential of the proposed COA system as an assistive tool in clinical practice. For future work, frequently collected data over extended periods can provide a deeper understanding of the variability of the disease, that is likely to contribute significantly to the variability of treatment response. Having larger and denser data sets will also assist in characterising intra- and inter-patient variability. It will be important in expanding this work to examine ataxia in children and in an increased number of diseases which cause CA. This study was the first step demonstrating the capabilities of objective measurement of CA and further research is required to understand the scope of applications, as well as limitations of this approach. This study utilized subjects who were able to complete all the tests listed, as support proof of concept for the distillation and combination of key instrumented metrics – the STAR concept. However, we aim to address this in a future study where this cohort will be recruited and tested as per a modified suite (standing test cannot be performed in non-ambulant subjects) of instrumented testing.

## Supplementary information

Supplementary Information.

## Data Availability

The dataset used and/or analysed during the current study available from the corresponding author on reasonable request.
