# Supplementary material for: Objective Assessment of Cerebellar Ataxia: A Comprehensive and Refined Approach
Source: Sci Rep. 2020 Jun 11;10:9493. doi: 10.1038/s41598-020-65303-7 (PMC7289865; doi:10.1038/s41598-020-65303-7)
Supplement: Supplementary file 1 — Supplementary Information. [file 41598_2020_65303_MOESM1_ESM.docx]

**Supporting Information**

Objective Assessment of Cerebellar Ataxia: A Comprehensive and Refined Approach

Bipasha Kashyap, Dung Phan, Pubudu N. Pathirana, Malcolm Horne, Laura Power and David Szmulewicz


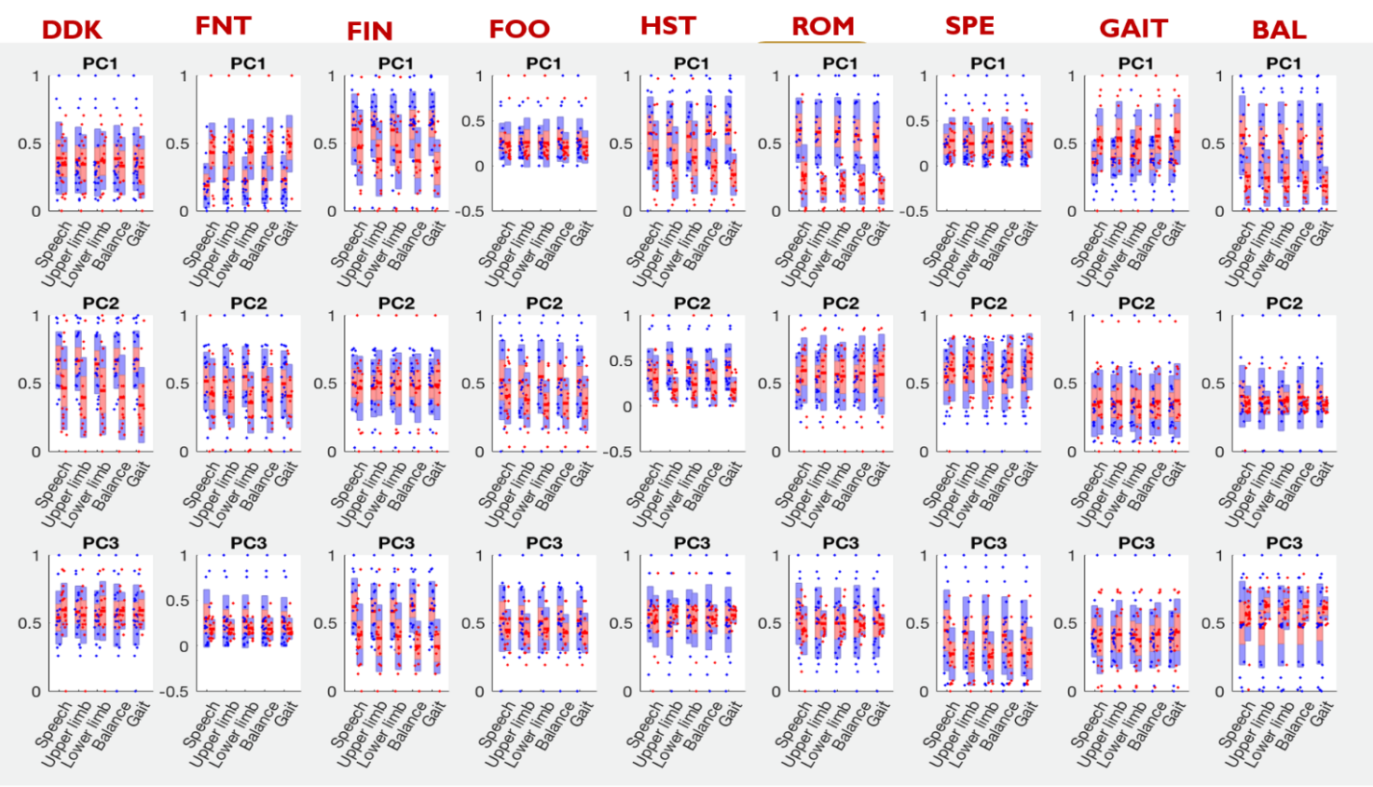


**Supplementary Figure S1.** Box plots demonstrating the distribution of the PCs 1-2-3 with respect to the 5 domains.


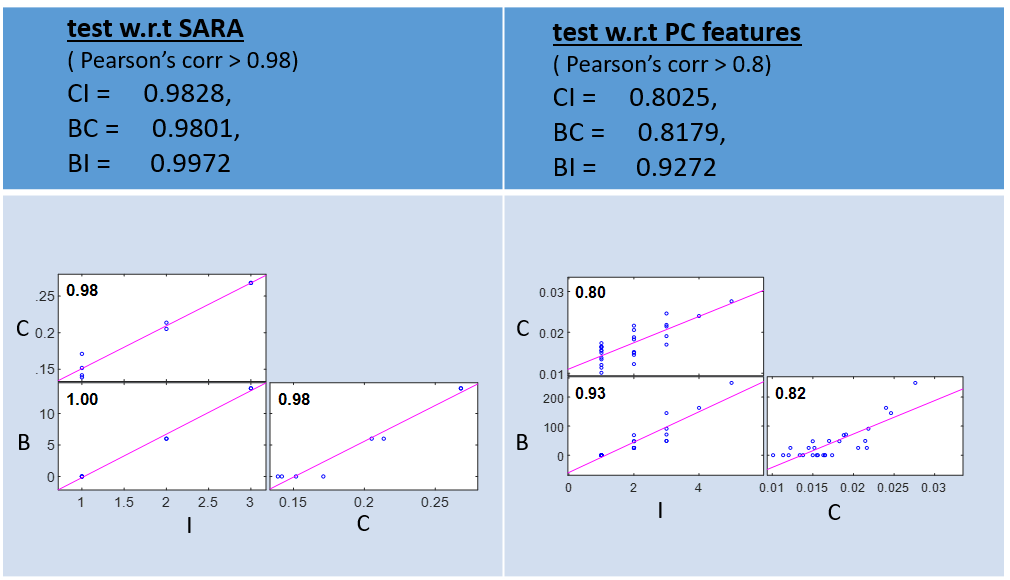


**Supplementary Figure S2.** The results of the centrality measures are highly correlated for both the MSTs. In addition, the corresponding rank orders based on our feature and SARA rating are similar.
